# Genetic Parameter Estimation of Body Weight and *Vp*_AHPND_ Resistance in Two Strains of *Penaeus vannamei*

**DOI:** 10.3390/ani15091266

**Published:** 2025-04-29

**Authors:** Guixian Huang, Jie Kong, Jiteng Tian, Sheng Luan, Mianyu Liu, Kun Luo, Jian Tan, Jiawang Cao, Ping Dai, Guangfeng Qiang, Qun Xing, Juan Sui, Xianhong Meng

**Affiliations:** 1 State Key Laboratory of Mariculture Biobreeding and Sustainable Goods, Yellow Sea Fisheries Research Institute, Chinese Academy of Fishery Science, Qingdao 266071, China; huangguixian0811@163.com (G.H.); kongjie@ysfri.ac.cn (J.K.); 0708jixiang@163.com (J.T.); luansheng@ysfri.ac.cn (S.L.); 2022213007@stu.njau.edu.cn (M.L.); luokun@ysfri.ac.cn (K.L.); tanjian@163.com (J.T.); caojw@ysfri.ac.cn (J.C.); daiping54@163.com (P.D.); qianggf1991@163.com (G.Q.); 2 Laboratory for Marine Fisheries Science and Food Production Processes, Qingdao Marine Science and Technology Center, Qingdao 266237, China; 3 College of Fisheries and Life Science, Shanghai Ocean University, Shanghai 201306, China; 4 College of Fisheries, Nanjing Agricultural University, Nanjing 210095, China; 5 BLUP Aquabreed Co., Ltd., Weifang 261311, China; xingqun527@163.com

**Keywords:** *Penaeus vannamei*, ssGBLUP, *Vp*_AHPND_, body weight

## Abstract

This study evaluated the genetic parameters for growth and *Vibrio parahaemolyticus* (*Vp*_AHPND_) resistance in both the introduced MK strain and the self-constructed GK strain of *Penaeus vannamei*, investigating the impact of genotyped female parents on trait estimates under a single-parent nested mating design. A total of 32 families from the MK strain and 44 families from the GK strain were analyzed. Fifty-four female parents from both strains were genotyped using the “Yellow Sea Chip No. 1” containing 10.0 K SNPs. In the MK strain, heritability estimates ranged from 0.439 to 0.458 for body weight (Bw) and from 0.308 to 0.489 for survival time (ST) and survival rates at 36 h (36 SR), 50% mortality (SS_50_), and 60 h (60 SR). In the GK strain, heritability for Bw ranged from 0.724 to 0.726, while ST, 36 SR, SS_50_, and 60 SR had heritability estimates between 0.370 and 0.593. Genetic correlations between Bw and ST were 0.601 to 0.622 in the MK strain and 0.742 to 0.744 in the GK strain. For Bw and survival rates, correlations ranged from 0.120 to 0.547 in the MK strain and from 0.426 to 0.906 in the GK strain. The genetic correlation between ST and survival rates was not significantly different from 1 (*p* > 0.05) in both strains. High Pearson correlations (0.853 to 0.997, *p* < 0.01) were observed among survival rates at different points. Predictive accuracies for Bw, ST, and survival rates using single-step genomic best linear unbiased prediction (ssGBLUP) were comparable to pedigree-based best linear unbiased prediction (pBLUP) in the MK strain, while in the GK strain, ssGBLUP improved predictive accuracies for Bw, ST, and SS_50_ by 0.20%, 0.32%, and 0.38%, respectively. The results indicate that both growth and *Vp*_AHPND_ resistance have significant breeding potential. Although the genetic correlation between weight and resistance varies across different populations, there is a positive genetic correlation between these traits, supporting the feasibility of multi-trait selection. To enhance genetic accuracy, breeding programs should include more genotyped progeny. These findings also suggest that infection frequency and observation time influence resistance performance and breeding selection, emphasizing the need for a tailored resistance evaluation program to improve breeding efficiency and reduce costs.

## Simple Summary

This study compared the genetic parameters of growth traits and resistance to acute hepatopancreatic necrosis disease (AHPND) in two strains of *Penaeus vannamei*. A controlled pathogen challenge test was conducted to evaluate five phenotypic traits: body weight, survival time post-infection, family survival rate at 36 h post-infection, family survival rate at the median lethal time within each strain, and family survival rate at 60 h post-infection. Two models were applied: the pedigree-based best linear unbiased prediction (pBLUP) model and the single-step genomic best linear unbiased prediction (ssGBLUP) model. Compared to pBLUP, ssGBLUP, which incorporates maternal genomic information, yielded higher heritability estimates for all traits in both strains. A moderate positive genetic correlation was observed between body weight and survival time under AHPND challenge, suggesting the feasibility of multi-trait selection for both growth and disease resistance. However, using only maternal genomic information provided limited improvement in the accuracy of genetic evaluations for body weight and resistance. To enhance breeding efficiency, future selection programs should incorporate genotyping data from a larger number of offspring.

## 1. Introduction

The Pacific white shrimp (*Penaeus vannamei*), native to the tropical coastal regions of Central and South America, achieved a global production of 6.8 million tons in 2022 [1], making it the most extensively farmed species worldwide. Introduced to China in 1988 [2], *P. vannamei* has flourished due to its high nutritional value, robust stress resistance, and suitability for high-density industrial farming. By 2023, the production of *P. vannamei* in China reached 2.23 million tons [3], accounting for approximately one-third of global output. In recent years, various shrimp diseases have severely impacted the global aquaculture industry, with acute hepatopancreatic necrosis disease (AHPND) being particularly notable. AHPND is caused by *Vibrio parahaemolyticus* (*Vp*_AHPND_) carrying a 69–73 kb toxin plasmid, leading to gut and hepatopancreas abnormalities in *P. vannamei*, and can result in mortality rates as high as 90% within 20–30 days post-infection [4,5,6]. Since its emergence in major shrimp farming regions in China, Southeast Asia, and South America in 2009, AHPND has led to annual losses exceeding USD 7 billion [4,7,8,9]. Although recognized as a notifiable aquatic animal disease by the World Organization for Animal Health (OIE) in 2016, a comprehensive management and control system for AHPND remains elusive.

Selective breeding offers a promising strategy for mitigating shrimp diseases [10], including the development of *P. vannamei* varieties resistant to *Vp*_AHPND_, which could potentially reduce the economic impact of this devastating disease [11]. Traditional methods, such as pedigree-based best linear unbiased prediction (pBLUP), rely on constructing a pedigree relationship matrix (A). However, this method has limitations when evaluating traits like disease resistance, which cannot be directly tested on candidate individuals and are often assessed using family siblings. This constraint results in the utilization of only 50% of the additive genetic variation present in the testing population, thereby diminishing prediction accuracy [12,13]. In contrast, genomic best linear unbiased prediction (GBLUP) uses genetic markers to construct a genetic relationship matrix (G) among test individuals. This approach allows for more precise capture of variations between full or half-siblings, effectively utilizing variance within or between populations and thus enhancing prediction accuracy [14]. Nevertheless, genetic marker typing incurs substantial costs.

The single-step genomic best linear unbiased prediction (ssGBLUP) method was introduced in 2009. ssGBLUP constructs a matrix (H) that integrates both pedigree and genomic relationships [15,16,17], enabling the accurate estimation of breeding values through genomic selection. This method is particularly beneficial for economically less valuable individuals. ssGBLUP has been widely applied in various aspects of aquaculture breeding, including enhancing disease resistance traits in rainbow trout [18], improving growth traits in *Macrobrachium rosenbergii* [19], optimizing weight characteristics in *Atlantic salmon* [20], and refining growth traits in *P. vannamei* [21].

Growth is the most economically significant trait in aquaculture. In China, thirteen of fifteen new varieties of *P. vannamei* are bred with growth as the target trait. To fully utilize existing germplasms and develop new varieties with both fast growth and *Vp*_AHPND_ resistance, this study aimed to estimate the genetic parameters of body weight and *Vp*_AHPND_ resistance in two *P. vannamei* strains. Furthermore, maternal genotypic data were used to conduct ssGBLUP and assess the evaluation accuracy.

## 2. Materials and Methods

### 2.1. Data Collection

#### 2.1.1. Experimental Shrimp

This study used non-endangered shrimp and did not involve genetically modified organisms. In accordance with the Fisheries Law of the People’s Republic of China, animal collection required no special permission, and no formal ethics approval was necessary.

This study involved two strains, designated as the MK strain and the GK strain. The MK strain, a commercial population known for its high survival rate in challenging environments, was introduced from the United States to BLUP Aquabreed Co., Ltd. in Weifang City, Shandong Province, China, in 2022. The GK strain consists of two foundational populations: the Primo brand, introduced from a USA-based company in Texas and recognized for its strong resistance to white spot syndrome virus (WSSV) [21], and a commercial population from Wenzhou, China, with an unclear genetic relationship to other strains. Both populations were collected in 2017 and are preserved by BLUP Aquabreed Co., Ltd. The breeding program for the GK strain aims to enhance WSSV resistance and growth rate through a multi-trait composite breeding technique, based on large-scale family selection. Generations G0 to G5 were established through single-parent nested mating.

In 2023, next-generation families were established for each strain using a single-parent nested mating design. Specifically, two mature females from different families within the same strain were placed into a 3 m^2^ tank with ten mature males from another family of the same strain. To establish a specific pathogen-free (SPF) nucleus, all broodstock shrimp were fed compound feed to induce maturation. In the single-parent nested mating design, natural mating of the broodstock was allowed, which helps improve the mating rate and offspring hatching rate. Following natural fertilization, the females were removed for ovulation and hatching in a separate 170 L bucket. Finally, a total of 76 families were established (32 MK families and 44 GK families). Approximately 20,000 nauplii from each family were randomly sampled and reared under standardized conditions, as Tan et al. [22] described. After two months, when the average body weight reached 2 g, fifty individuals from each family were randomly selected and transferred to the Yellow Sea Fisheries Research Institute for further rearing and assessment of body weight and *Vp*_AHPND_ resistance. Pathogen screening was conducted on three randomly selected individuals per family, including one bacterial pathogen (*Vp*_AHPND_) and three viral pathogens: WSSV, Taura syndrome virus (TSV), and infectious hypodermal and hematopoietic necrosis virus (IHHNV). The results confirmed that none of these pathogens were present. In total, 960 individuals from the MK strain and 1320 individuals from the GK strain were included in the genetic parameter assessment. The MK strain test individuals were aged P62–P67, while the GK strain test individuals were aged P45–P74.

#### 2.1.2. Body Weight and *Vp_AHPND_* Resistance Test

The experiment was conducted in four tanks, each equipped with an identical recirculating aquaculture system [23]. Forty shrimp were randomly selected from each family and evenly distributed into 4 groups (1 control group and 3 experimental groups), with each group placed in a different tank. Every group of 10 shrimp was housed in a 10 cm × 15 cm × 35 cm rectangular grid box, with the shrimp separated by perforated plastic dividers.

The *Vp*_AHPND_ strain used in this experiment was obtained from the Third Institute of Oceanography, Ministry of Natural Resources, and stored at −80 °C. A preliminary experiment was conducted to determine that, for *P. vannamei* weighing 2–3 g, the median lethal dose (LD50) of *Vp*_AHPND_ infection within 60 h is 10^7^ colony forming unit (CFU). A bacterial suspension of *V. parahaemolyticus* at a concentration of 10^9^ CFU/mL was prepared. Commercial pellet feed was immersed in this suspension for 50 min at 28 °C. According to the plate counting method, each pellet of feed contained 4.71–9.43 × 10^6^ CFU of the pathogen [24]. Prior to the experiment, the shrimp underwent a 24 h fasting period to ensure their gastrointestinal tracts were empty.

The experimental shrimp were fed 7 pellets of poisoned feed at 0 and 36 h, ensuring that each shrimp ingested a consistent amount of *V. parahaemolyticus*, reaching 10^7^ CFU during each feeding. The control group was fed commercial pellet feed soaked in a culture medium that was not inoculated with *V. parahaemolyticus*. Observations were conducted every 6 h following the initial feeding of contaminated pellets. Deceased shrimp were promptly removed, and data including body weight (Bw), family ID, and survival time (ST: the time from the initiation of toxic pellet feeding at 0 h to the death of the shrimp) were recorded. For individuals that survived until the end of the experiment, ST was recorded as the experiment’s endpoint. The experiment was terminated upon reaching a cumulative mortality rate of 60% within each strain. The experiment lasted for 60 h. Survival rates for each family within each strain were recorded at three time points: the survival rate of each family at 36 h (36 SR), the survival rate of each family when the mortality within the strain reached 50% (SS_50_), and the survival rate of each family at 60 h (60 SR). Throughout the experiment, seawater temperature was maintained at 27–28 °C with a salinity of 33‰. Commercial pellet feed was administered at 10:00 AM and 10:00 PM, with each feeding session comprising 12% of the shrimp’s body weight. Each morning, remaining pellets and fecal matter were removed from the tanks, and 40% of the water was replaced. No mortality was observed in the control group during the infection testing period.

### 2.2. Genotyping

A total of 54 female parents were genotyped using the 10K SNP “Yellow Sea Chip No. 1”, including 23 individuals from the MK strain and 31 from the GK strain. Initially, 10,416 SNP loci were identified. After quality control using Plink v1.9 (MAF < 0.05, SNP missing rate > 0.1, individual missing rate > 0.2), 10,307 SNPs remained for subsequent analysis.

### 2.3. Data Analysis

#### 2.3.1. Construction of the Relationship Matrix

A single-parent nested mating design was used to construct the families. This approach allows for the precise identification of maternal individuals within each family. In contrast, paternal individuals are not directly identifiable and are considered virtual. They are inferred to belong to the male shrimp families. Pedigree reconstruction is subsequently facilitated by leveraging information from this male family. For the MK strain, pedigree relationships were traced from the G0 and G1 generations between 2022 and 2023, including a total of 1045 individuals. For the GK strain, pedigree relationships extended from the G0 to G6 generations between 2018 and 2023, including 1935 individuals. The A matrix was constructed using the ASReml-R V4.1 software package [25]. The H matrix, integrating both pedigree and genotype information, was constructed using the preGSf90 program within the BLUPF90 1.70 package [26].

#### 2.3.2. Estimation of Genetic Parameters

Variance components for Bw and ST were estimated using the average information restricted maximum likelihood (REML) method. This approach utilized the relationship matrices (A and H) and an animal model implemented in the ASReml-R V4.1 package [25]. The animal model is specified as follows:

$$y_{Bwi}=\mu_{Bw}+Age_{i}+a_{i}+e_{Bwi},$$

$$y_{STi}=\mu_{ST}+Bw_{i}+a_{i}+e_{STi},$$

where $y_{Bwi}$ and $y_{STi}$ are the observed values of Bw and ST for the ith individual; $\mu_{Bw}\;$ and $\mu_{ST}$ are the means of Bw and ST; $Age_{i}$ represents the age in days of the ith individual (covariate)$;\;Bw_{i}$ is the body weight of the ith individual (covariate); $a_{i}$ is the additive genetic effects for the ith individual; and $e_{Bwi}$ and $e_{STi}$ are the random residual effects for Bw and ST of the ith individual.

The phenotype variance is the sum of all variance components and is calculated using the following formula:

$$\sigma_{p}^{2}=\sigma_{a}^{2}+\sigma_{e}^{2},$$

Heritability ($h^{2}$) is the ratio of additive genetic variance to phenotypic variance, and is calculated using the following formula:

$$h^{2}=\sigma_{a}^{2}/\sigma_{p}^{2},$$

where $\sigma_{p}^{2}$ is the phenotypic variance, $\sigma_{a}^{2}$ is the additive variance, and $\sigma_{e}^{2}$ is the residual variance component.

Genetic parameters for survival rates (36 SR, SS_50_, and 60 SR) were estimated using the ASReml-R V4.1 software package [25] with a parental threshold trait model and a Probit link function. In this model, at 36 h, individuals that died were recorded as 0, and those that survived were recorded as 1. When the number of dead individuals reached half of the total population, the dead individuals were recorded as 0, and survivors as 1. The same recording system was applied at 60 h. The model is as follows:

$$\mathrm{Pr}\left(y_{ijkg}=1\right)=\mathrm{Pr}\left(l_{ijkg}>0\right)=\Phi \left(\mu {+Tank_{g}+s}_{i}+d_{j}+e_{ijkg}\right),$$

where Pr represents the probability of an individual surviving; $y_{ijkg}$ is the survival status of the kth individual (0: deceased; 1: survived); $l_{ijkg}$ is the latent variable for $y_{ijkg}$ (if $l_{ijkg}>0$, then $y_{ijkg}=1$; if $l_{ijkg}\leq 0$, then $y_{ijkg}=0$); μ is the overall mean; $Tank_{g}$ is the fixed effect of the specific tank number; $S_{i}$ is the additive genetic effects of the ith sire; $d_{j}$ is the additive genetic effects of the jth dam; and $e_{ijkg}$ is the random residual for the kth individual.

The heritability ($h_{u}^{2}$) of 36 SR, SS_50_, and 60 SR is calculated using the following formula:

$$h_{u}^{2}=4\sigma_{sd}^{2}/(2\sigma_{sd}^{2}+\sigma_{e}^{2}),$$

The heritability ($h_{p}^{2}$) on the observed scale is calculated using the following formula:

$$h_{p}^{2}=h_{u}^{2}\times z^{2}/(p(1-p)),$$

where p is the proportion of surviving individuals among all individuals, and z is the ordinate of the highest point of the normal distribution.

#### 2.3.3. Genetic Correlation

A bivariate animal model was employed to estimate both the phenotypic and genetic correlations between different traits:

$$r_{gij}=cov\left(\sigma_{i},\sigma_{j}\right)/\sigma_{ai}\sigma_{aj},$$

where $r_{gij}$ is the genetic correlations between trait i and trait j; $cov(\sigma_{i},\sigma_{j})$ is the covariances between trait i and trait j; and $\sigma_{ai}$ and $\sigma_{aj}$ are the additive genetic standard deviations for trait i and trait j, respectively.

#### 2.3.4. Z-Test

Z-scores were used to assess the significance of differences in heritability and genetic correlation estimates.

$$Z=(X_{i}-X_{j})/\sqrt{\sigma_{i}^{2}+\sigma_{j}^{2}},$$

where $X_{i}$ and $X_{j}$ are the parameter estimates, and $\sigma_{i}^{2}$ and $\sigma_{j}^{2}$ are their standard errors. To test significance against 0 or 1, $X_{j}$ and $\sigma_{j}$ were set accordingly. A Z-score ≥ 1.96 indicates significance; ≥2.58 indicates high significance [27].

#### 2.3.5. Cross-Validation

To compare the prediction accuracies of pBLUP and ssGBLUP, five-fold cross-validation was performed with ten replicates to reduce sampling variability. In each run, one subset served as the validation set, and the remaining four as the reference set. Predictive accuracy was assessed by Pearson’s correlation between EBV (or GEBV) and phenotypes, while prediction bias was evaluated by the regression coefficient of phenotypes on EBV (or GEBV), with a value of 1 indicating unbiased estimates [28].

## 3. Results

### 3.1. Cumulative Mortality

**Figure 1:**
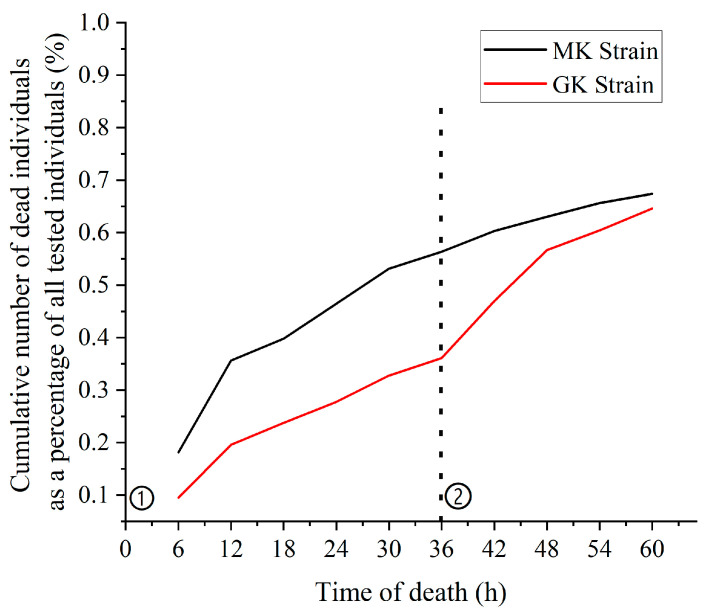
Cumulative mortality curves of MK and GK strains infected with *Vp*_AHPND_. Notes: Blackline: MK strain; red line: GK strain. ① the first infection at 0 h; ② the second infection at 36 h.

Following the initial feeding of poisoned pellets (0 h), both the MK and GK strains exhibited a rapid increase in mortality rates between 6 and 12 h, with the MK strain showing a higher mortality rate compared to the GK strain (Figure 1). After the second feeding of poisoned pellets (36 h), the mortality rate in the MK strain did not increase significantly, while the GK strain experienced a brief peak in mortality. By 30 h, the morality rate of the MK strain had reached 50%, while the GK strain reached the same rate at 48 h. Over the 60 h observation period, the cumulative mortality rates were 67.40% for the MK strain and 64.55% for the GK strain, with the MK strain demonstrating a slightly higher overall mortality rate.

### 3.2. Descriptive Statistics

Descriptive statistics for Bw, ST, and survival rates (36 SR, SS_50_, and 60 SR) for both strains are presented in Table 1. Descriptive statistics indicated that the two strains exhibited comparable body sizes (MK: 2.10 g; GK: 2.14 g), with slightly higher variability in the GK strain (CV = 20.92%) than in the MK strain (CV = 15.58%). For survival-related traits, the GK strain demonstrated superior resistance to *Vp*_AHPND_, with a longer average survival time (GK: 31.64 h; MK: 23.62 h) and higher survival rates at 36 and 60 h post-infection (36 SR: GK 63.94% vs. MK 43.65%; 60 SR: GK 35.30% vs. MK 32.71%) compared to the MK strain. In addition, the GK strain showed lower coefficients of variation for ST, 36 SR, and 60 SR, indicating more stable phenotypic expression of disease resistance. Notably, 60 SR had the highest variability in both strains, suggesting substantial individual differences and potential for further genetic improvement.

**Figure 2:**
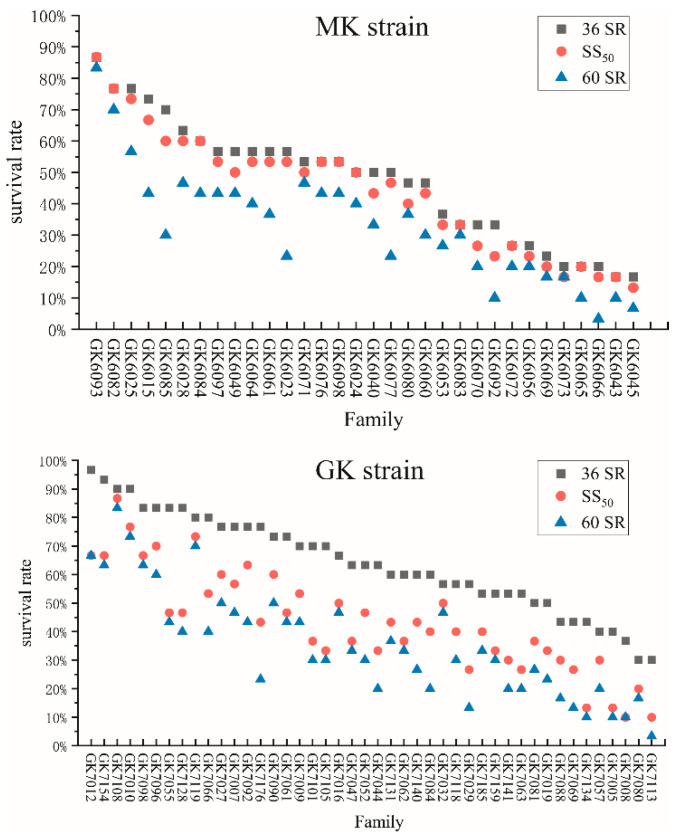
Survival rates of all families from both strains at different time points. Notes: ■ 36 SR: the survival rate of each family at 36 h; 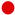 SS_50_: the survival rate of each family when the mortality within the strain reached 50%; and 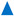 60 SR: the survival rate of each family at 60 h.

Survival rates of test families for *Vp*_AHPND_ resistance at different time points are shown in Figure 2. The results showed that the survival correlations at the three time points ranged from 0.853 to 0.997 (*p* < 0.01) (Table 2). In the MK strain, the 36 SR and SS_50_ time points exhibited similar survival rate trends, while in the GK strain, the SS_50_ and 60 SR time points showed similar survival rate trends.

### 3.3. Molecular Genetic Correlation Analysis

**Figure 3:**
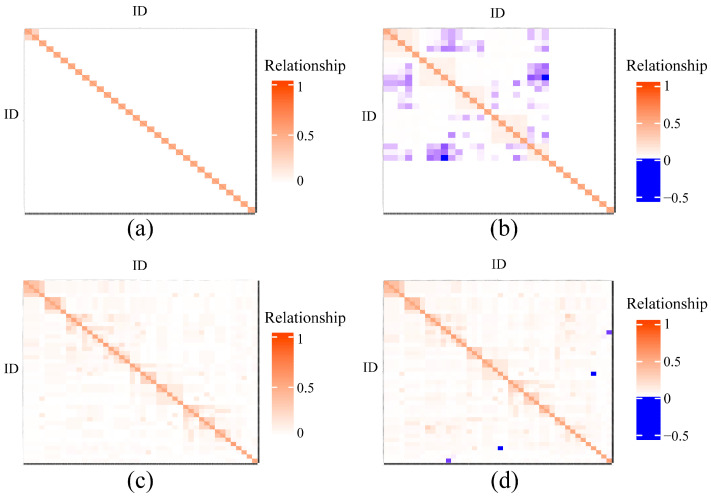
Heatmap of genetic relationships for the two strains based on different matrices. Notes: (**a**) the relationship heatmap of the A matrix for the MK strain; (**b**) the relationship heatmap of the H matrix for the MK strain; (**c**) the relationship heatmap of the A matrix for the GK strain; and (**d**) the relationship heatmap of the H matrix for the GK strain.

The heatmaps illustrating the relationships between individuals in the two strains, based on the A matrix and H matrix, are shown in Figure 3. In the MK strain, the kinship coefficient correlation between the off-diagonal elements of both matrices was 0.969. However, due to the limited pedigree in the MK strain, which consists of only two generations, the relationship between the parents is zero, resulting in no standard deviation and preventing the calculation of the correlation coefficient for the diagonal elements. In contrast, the GK strain showed a kinship coefficient correlation of 0.608 between the diagonal elements of the two matrices, while the correlation between the off-diagonal elements was 0.994.

### 3.4. Heritability Estimation

The variance components and heritability estimate for Bw, ST, and survival rates (36 SR, SS_50_, and 60 SR) in the two strains are presented in Table 3. The heritability estimates based on matrix A were 0.439 ± 0.108 and 0.726 ± 0.119 for Bw in the MK and GK strains, respectively. For the resistance traits, ST and survival rates (36 SR, SS_50_, and 60 SR), the MK strain exhibited heritability estimates ranging from 0.308 ± 0.079 to 0.443 ± 0.108, while the GK strain showed estimates between 0.372 ± 0.084 and 0.572 ± 0.116. Based on matrix H, heritability estimates for Bw, ST, and survival rates (36 SR, SS_50_, and 60 SR) in the MK strain increased by 4.56%, 7.47%, 9.93%, 12.93%, and 9.79%, respectively. In the GK strain, the heritability of survival rates (36 SR, SS_50_, and 60 SR) increased by 3.23% to 5.14%, whereas the estimates for Bw and ST slightly decreased by 0.28% and 0.54%, respectively. Z-score tests revealed highly significant differences in the heritability between 60 SR of the MK and GK strains obtained by both methods (*p* < 0.01).

### 3.5. Genetic Correlations

Genetic and phenotypic correlations between traits, estimated using different methods for both strains, are presented in Table 4 and Table 5. In the MK strain, the genetic correlation between ST and Bw was moderate (0.601 with pBLUP; 0.622 with ssGBLUP), while the correlations between Bw and survival rates (36 SR, SS_50_, and 60 SR) ranged from low to moderate (0.120–0.547). In contrast, the GK strain exhibited stronger positive genetic associations between growth and resistance traits, with ST and Bw showing high correlations (0.742 with pBLUP; 0.744 with ssGBLUP), and survival rates showing moderate to very high correlations with Bw (0.426–0.906). These results suggest that in the GK strain, selection for increased growth is more likely to concurrently improve disease resistance traits, thereby facilitating multi-trait genetic improvement. The genetic correlations between ST and SS_50_ were very high in both strains, with no significant difference from 1 (*p* > 0.05). Furthermore, the genetic correlations between survival rates at different time points were all greater than 0.962, indicating a high positive correlation, but these results are not presented here for brevity.

### 3.6. Predictive Accuracy and Bias Analysis

The results of predictive accuracy and bias analysis for Bw, ST, and survival rates (36 SR, SS_50_, and 60 SR) obtained using five-fold ten-times cross-validation, are presented in Table 6. The results showed that the predictive accuracies for Bw, ST, and survival rates (36 SR, SS_50_, and 60 SR) using ssGBLUP were comparable to those obtained using pBLUP in the MK strain. In the GK strain, predictive accuracies for Bw, ST, and SS_50_ were improved by 0.20%, 0.32%, and 0.38%, respectively, with ssGBLUP compared to pBLUP.

## 4. Discussion

### 4.1. Genetic Heritability of Body Weight and Predictive Accuracy

Body weight is the most important economic trait in *P. vannamei*. Numerous studies have been conducted to estimate the genetic parameters of body weight in this species, with heritability estimates ranging from 0.13 to 0.81 for pBLUP and from 0.17 to 0.59 for ssGBLUP [29,30,31]. Tan [22] and Luan [32] evaluated the heritability of body weight in *Penaeus vannamei* under different stocking densities, with values ranging from 0.26 to 0.44. Trang [33] estimated the heritability of body weight in *P. vannamei* after 120 days of culture to be 0.16. Fu [21] assessed the heritability of body weight in *P. vannamei* under a hybrid strategy for growth and WSSV resistance, reporting a value of 0.339. Sui [34] evaluated the heritability of body weight in *P. vannamei* at the time of WSSV-induced mortality, with values ranging from 0.453 to 0.810. In this study, we estimated the heritability of body weight in two strains of *P. vannamei*, and the results showed that both pBLUP (MK: 0.439; GK: 0.726) and ssGBLUP (MK: 0.458; GK: 0.724) yielded heritability estimates higher than those reported in most previous studies. An important reason for this is the exclusion of the common environment effect, which is crucial for accurately estimating heritability [35,36]. The common environment effect results from the separate rearing of full-sib families prior to testing, as well as one-quarter of the non-additive genetic effect shared by full-sibs, in the model. Because each family was reared separately both before and after the *Vp*_AHPND_ infection, the common environmental effects were confounded with additive genetics when estimating the variance components, which could not be effectively separated by the model, leading to an overestimation of heritability [37,38,39,40,41].

The heritability of body weight in the GK strain was higher than that in the MK strain (*p* < 0.05). This difference may be attributed to one main factor: the genetic background. The MK strain originated from a commercial population, which typically exhibits very narrow genetic diversity, and we were only able to determine their relationships through a one-generation pedigree. In contrast, the GK strain has a diverse genetic background, derived from high-quality, disease-resistant germplasms. Even though the strain was primarily selected for WSSV resistance and growth traits, the weight assigned to growth in the selection index was relatively small (only 25%). Therefore, even after five generations of selection, the genetic diversity related to growth has largely been retained.

The pedigree heatmap results derived from the A and H matrices indicated that incorporating maternal genomic information had a marginal effect on improving pedigree relationships in this study. One possible explanation is that the family structure was based on a single-parent mating design, where the paternal parent could only be traced to the family level. Genotyping only the female parents does not resolve the uncertainty regarding the origin of the male parents. This issue is particularly apparent in the MK population, which has only two generations of pedigree. Furthermore, the inclusion of maternal genomic information had a minimal impact on enhancing prediction accuracy [42,43]. The prediction accuracy results in this study showed that ssGBLUP, incorporating maternal genomic information, yielded comparable accuracy to pBLUP for most traits in both strains. Notably, slight improvements were observed in the GK strain for Bw, ST, and SS_50_, with increases ranging from 0.20% to 0.38%. Liu et al. [44] and Garcia et al. [45] suggest that full-sibling cross-validation may be more suitable for evaluating predictive accuracy, as it requires both phenotypic and genotypic data from the test individuals. Offspring phenotypes may not fully reflect the parental genotypic expression, potentially introducing genetic variability and reducing the accuracy of heritability estimates. To optimize genomic estimated breeding values (GEBVs), Van et al. [46] recommend measuring both phenotypic and genotypic data for the same individuals, especially when phenotypic data are limited. Therefore, in breeding programs based on a single-parent mating design, it is advisable to prioritize genotyping individuals with available phenotypic records. Additionally, genotyping individuals with extreme phenotypes (e.g., the highest and lowest values for body weight or disease resistance) can be considered. Such a genotyping strategy not only reduces genotyping costs but also enhances the accuracy of pedigree assignment and genomic prediction [47]. The accuracy of breeding value predictions is also influenced by factors such as the reference population size, selection of genotyped individuals, number of genotyped individuals, number of effective SNP loci post-genotyping, and the model used for validation [48,49,50,51,52,53,54,55].

### 4.2. Genetic Heritability of Vp_AHPND_ Resistance and Predictive Accuracy

*Vp*_AHPND_ has caused significant losses in *P. vannamei* aquaculture, prompting researchers to evaluate the genetic parameters for resistance to *Vp*_AHPND_ in this species using various testing methods. Wang et al. [56] estimated the heritability of *Vp*_AHPND_ resistance using an injection method, with pBLUP estimates ranging from 0.15 to 0.24, and slightly higher estimates from GBLUP (0.16–0.26). Similarly, Lyu et al. [57], also employing the injection method, reported heritability estimates based on pedigree relationships ranging from 0.12 to 0.23, with molecular-based estimates (0.10 to 0.20) showing similar trends. Liu et al. [58] and Liu et al. [28] used the immersion method for *Vp*_AHPND_ infection, reporting varying heritability estimates. Liu et al. [28] found a heritability estimate of 0.24 ± 0.07 using ssGBLUP, while Liu et al. [58] reported higher estimates (pBLUP: 0.79; GBLUP: 0.68; ssGBLUP: 0.75). In existing studies, *Vp*_AHPND_ infection is commonly induced by injection or immersion. However, improper injection can cause stress-induced mortality in shrimp, while immersion makes it difficult to determine the exact infection dose per individual. Oral challenge using pathogen-laced feed can overcome both of these limitations. Huang et al. [59], using toxic feed for infection, reported heritability estimates based on pedigree data ranging from 0.17 to 0.37. In the present study, using toxic feed for infection, the heritability estimates (pBLUP: 0.308–0.572; ssGBLUP: 0.331–0.593) were higher than those in previous studies (pBLUP: 0.12–0.37; ssGBLUP: 0.11–0.26). Similarly to growth traits, failure to account for common environmental effects may contribute to these differences. Furthermore, variations in infection methods and experimental populations from different sources may also explain the observed discrepancies.

The resistance differences between the two populations were also notable. The heritability of resistance to *Vp*_AHPND_ in the GK strain (0.370–0.593) was higher than in the MK strain (0.308–0.489), which may be associated with the genetic diversity within the populations. The MK strain reached 50% mortality within 30 h, whereas the GK strain required 48 h to reach the same level. The SS_50_ data for the MK strain reflect the outcome after a single dose of toxic feed, while the SS_50_ data for the GK strain represent the result after two doses. This explains the higher average SS_50_ value for the MK strain compared to the GK strain. These findings are also consistent with the trends shown in Figure 2, where the MK strain exhibits more similar trends between 36 SR and SS_50_, whereas the GK strain shows closer alignment between SS_50_ and 60 SR. In this regard, survival time post-infection may serve as a better indicator for assessing the level of resistance differences between populations. It is also worth noting that previous studies suggest a potential positive genetic correlation between different resistance traits. Campos-Montes et al. [60] evaluated the genetic correlation between resistance to AHPND and WSSV survival rate in two strains of *P. vannamei*, finding a moderate to low positive correlation. Similarly, Lu et al. [27] identified a positive correlation between WSSV resistance and acute ammonia nitrogen stress in *P. vannamei*. The GK strain has been selected for both WSSV resistance and rapid growth over five generations; whether breeding for WSSV resistance also improves resistance to *Vp*_AHPND_ in *P. vannamei* requires further investigation.

Additionally, Pearson correlation analysis of the survival rates at 36 h, SS_50_, and 60 h across families showed correlation coefficients between 0.853 and 0.997 (*p* < 0.01) at the three time points. These results suggest that the performance of families after a single exposure to toxic feed is likely to reflect their resistance level, indicating that a second exposure may not be necessary. It is noteworthy that the survival rate rankings of families at different time points post-infection were highly correlated in both populations. This suggests that, when designing breeding schemes for resistance testing, the timing of survival assessments could be appropriately advanced to reduce labor and time costs, thereby improving testing efficiency.

### 4.3. Correlation Analysis Between Body Weight and Vp_AHPND_ Resistance

Understanding the relationship between body weight and disease resistance is crucial for developing effective breeding programs. Genetic correlations ($r_{g}$) are generally classified as high ($r_{g}$ > 0.8), moderate (0.4 < $r_{g}$ < 0.8), or low ($r_{g}$ < 0.4) [36,61,62]. The genetic correlation between growth and disease resistance varies significantly across different populations and pathogens. For instance, Argue et al. [63] reported a negative genetic correlation (−0.46) between growth and resistance to Taura syndrome, while Feng [64] observed a positive genetic correlation (0.69) between body weight and resistance to WSSV in 4-month-old *P. vannamei*. In contrast, Sui et al. [34] found a low negative genetic correlation (−0.198 to −0.019) between body weight and WSSV resistance. Growth and resistance traits are influenced by multiple genes, and the interactions between these genes can lead to varying degrees of correlation between different traits [65]. Research on the genetic correlation between growth and *Vp*_AHPND_ resistance in *P. vannamei* remains scarce. Huang et al. [59] are some of the few to report a positive genetic correlation (0.061 to 0.235) between body weight and *Vp*_AHPND_ resistance in high-WSSV resistant and fast-growth strains. Lyu et al. [57] reported a strong positive genetic correlation (0.98 to 0.99) between survival time and semi-lethal survival rate following *Vp*_AHPND_ infection. In this study, the GK strain showed much higher estimates for the correlation between body weight and survival rates (36 SR, SS_50_, and 60 SR) compared to the MK strain. This discrepancy may be due to differences in the timing of toxic feed administration (at 0 and 36 h). The MK strain reached 50% mortality before the second feeding (30 h), while the GK strain did not reach this point until 48 h. This difference in mortality timing likely amplified the correlation between body weight and *Vp*_AHPND_ resistance, potentially affecting the data structure for survival rate and influencing genetic parameter estimates.

## 5. Conclusions

This study provides several key findings regarding genetic potential and breeding strategies for improving body weight and *Vp*_AHPND_ resistance in commercial and multi-generational breeding populations. First, both body weight and *Vp*_AHPND_ resistance show strong genetic potential for selection in the evaluated populations, indicating that these traits can be effectively improved through breeding programs. Second, the genetic correlations between body weight and various metrics of *Vp*_AHPND_ resistance (survival time and survival rates at different time points) exhibited varying degrees of positive correlation, demonstrating that both traits can be improved simultaneously without compromising each other. Third, under a single-parent nested mating design, using genotyping data from female parents alone resulted in limited improvement in prediction accuracy. This highlights the need for expanded genotyping of offspring to enhance breeding precision. Four, infection frequency and observation intervals significantly influence the observed resistance performance and subsequent selection outcomes. This emphasizes the importance of implementing targeted resistance evaluation strategies to enhance breeding efficiency and reduce costs. In summary, this study underscores the importance of integrating comprehensive genotyping and targeted resistance evaluation strategies into breeding programs to effectively improve both body weight and *Vp*_AHPND_ resistance. Future research should focus on expanding genotyping efforts and optimizing resistance evaluation protocols to further enhance breeding outcomes.

## Figures and Tables

**Table 1:** Descriptive statistics for Bw, ST, and survival rates at 3 time points in both MK and GK strains.

Strain	Trait	Mean	Max	Min	SD	CV
MK	Bw	2.10 g	2.76 g	1.17 g	0.33 g	15.58%
	ST	23.62 h	35.36 h	12.89 h	6.14 h	25.98%
	36 SR	43.65%	86.67%	13.33%	19.12%	43.80%
	SS_50_	46.88%	86.67%	16.67%	19.11%	40.77%
	60 SR	32.71%	83.33%	3.33%	17.73%	54.19%
GK	Bw	2.14 g	2.95 g	1.39 g	0.45 g	20.92%
	ST	31.64 h	42.6 h	19.44 h	5.56 h	17.58%
	36 SR	63.94%	96.67%	30.00%	12.75%	19.94%
	SS_50_	43.33%	86.67%	10.00%	13.09%	30.20%
	60 SR	35.30%	83.33%	3.33%	12.05%	34.13%

Notes: Bw: body weight; ST: the time from the initiation of toxic pellet feeding at 0 h to the death of the shrimp; 36 SR: the survival rate of each family at 36 h; SS_50_: the survival rate of each family when the mortality within the strain reached 50%; and 60 SR: the survival rate of each family at 60 h.

**Table 2:** Pearson correlation of survival rates at different time points.

Strain	MK			GK		
Mortality Rate	36 SR	SS_50_	60 SR	36 SR	SS_50_	60 SR
36 SR	1	-	-	1	-	-
SS_50_	0.977 ^a^	1	-	0.892 ^a^	1	-
60 SR	0.884 ^a^	0.869 ^a^	1	0.853 ^a^	0.951 ^a^	1

Notes: 36 SR: the survival rate of each family at 36 h; SS_50_: the survival rate of each family when the mortality within the strain reached 50%; and 60 SR: the survival rate of each family at 60 h. The lower triangle represents correlation, ^a^: indicates that the statistical test reached the highly significant level (*p* < 0.01).

**Table 3:** Variance components and heritability estimates for Bw, ST, and survival rates (36 SR, SS_50_, and 60 SR) were evaluated in both MK and GK strains.

Method	Trait	MK			GK		
		$\sigma_{a}^{2}$	$\sigma_{e}^{2}$	$h^{2}$	$\sigma_{a}^{2}$	$\sigma_{e}^{2}$	$h^{2}$
pBLUP	Bw	0.443	0.035	0.439 ± 0.108	0.661	0.064	0.726 ± 0.119
	ST	278.997	16.721	0.308 ± 0.079	342.742	20.352	0.372 ± 0.084
	36 SR	1.284	0.089	0.443 ± 0.108 ^ab^	1.338	0.099	0.505 ± 0.111 ^ab^
	SS_50_	1.277	0.087	0.433 ± 0.106 ^ab^	1.329	0.097	0.496 ± 0.109 ^ab^
	60 SR	1.273	0.088	0.429 ± 0.108 ^a^	1.401	0.114	0.572 ± 0.116 ^b^
ssGBLUP	Bw	0.448	0.037	0.458 ± 0.111	0.660	0.064	0.724 ± 0.119
	ST	282.922	17.601	0.331 ± 0.083	342.392	20.269	0.370 ± 0.084
	36 SR	1.249	0.102	0.487 ± 0.116 ^ab^	1.306	0.105	0.531 ± 0.115 ^ab^
	SS_50_	1.251	0.101	0.489 ± 0.117 ^ab^	1.301	0.102	0.512 ± 0.113 ^ab^
	60 SR	1.214	0.099	0.471 ± 0.116 ^a^	1.371	0.118	0.593 ± 0.119 ^b^

Notes: Bw: body weight; ST: the time from the initiation of toxic pellet feeding at 0 h to the death of the shrimp; 36 SR: the survival rate of each family at 36 h; SS_50_: the survival rate of each family when the mortality within the strain reached 50%; and 60 SR: the survival rate of each family at 60 h. $\sigma_{a}^{2}$ is additive genetic variance; $\sigma_{e}^{2}$ is residual variance; $\sigma_{p}^{2}$ is phenotypic variance; and $h^{2}$ is the heritability. Different letters indicate a highly significant difference in survival between the two strains under the same methodology (*p* < 0.01).

**Table 4:** Variance components and heritability estimates for Bw, ST, and survival rates (36 SR, SS_50_, and 60 SR) were evaluated in MK strain.

Method	Trait	MK				
		Bw	ST	36 SR	SS_50_	60 SR
pBLUP	Bw	-	0.126 ± 0.055 ^b^	0.175 ± 0.074 ^b^	0.068 ± 0.077 ^a^	0.461 ± 0.101
	ST	0.601 ± 0.155	-	0.966 ± 0.001 ^c^	0.976 ± 0.001 ^c^	0.982 ± 0.002 ^c^
	36 SR	0.287 ± 0.125 ^b^	0.998 ± 0.000 ^c^	-	0.899 ± 0.007	0.870 ± 0.011
	SS_50_	0.120 ± 0.139 ^a^	0.995 ± 0.001 ^c^	0.975 ± 0.012 ^c^	-	0.949 ± 0.013 ^c^
	60 SR	0.503 ± 0.297	0.988 ± 0.002 ^c^	0.991 ± 0.006 ^c^	0.959 ± 0.011 ^c^	-
ssGBLUP	Bw	-	0.179 ± 0.056	0.101 ± 0.122 ^a^	0.088 ± 0.120 ^a^	0.454 ± 0.267 ^a^
	ST	0.622 ± 0.145	-	0.954 ± 0.007 ^c^	0.971 ± 0.003 ^c^	0.987 ± 0.000 ^c^
	36 SR	0.169 ± 0.189 ^a^	0.955 ± 0.009 ^c^	-	0.886 ± 0.005	0.931 ± 0.008 ^c^
	SS_50_	0.150 ± 0.187 ^a^	0.994 ± 0.002 ^c^	0.994 ± 0.004 ^c^	-	0.859 ± 0.005
	60 SR	0.547 ± 1.011 ^a^	0.999 ± 0.000 ^c^	0.992 ± 0.003 ^c^	0.993 ± 0.004 ^c^	-

Notes: Bw: body weight; ST: the time from the initiation of toxic pellet feeding at 0 h to the death of the shrimp; 36 SR: the survival rate of each family at 36 h; SS_50_: the survival rate of each family when the mortality within the strain reached 50%; and 60 SR: the survival rate of each family at 60 h. Phenotypic correlation (above the diagonal) and genetic correlation (under the diagonal). ^a^: estimates are not significantly different from zero (*p* > 0.05); ^b^: estimates are significantly different from zero (*p* < 0.05); and ^c^: estimates are not significantly different from one (*p* > 0.05).

**Table 5:** Genetic and phenotypic correlations of Bw, ST, and survival rates (36 SR, SS_50_, and 60 SR) based on different methods in GK strain.

Method	Trait	GK				
		Bw	ST	36 SR	SS_50_	60 SR
pBLUP	Bw	-	0.348 ± 0.050	0.359 ± 0.017	0.775 ± 0.063	0.789 ± 0.019
	ST	0.742 ± 0.091	-	0.919 ± 0.003 ^c^	0.994 ± 0.001 ^c^	0.921 ± 0.006 ^c^
	36 SR	0.426 ± 0.032	0.999 ± 0.000 ^c^	-	0.789 ± 0.008	0.745 ± 0.005
	SS_50_	0.785 ± 0.064	0.997 ± 0.001 ^c^	0.979 ± 0.007 ^c^	-	0.781 ± 0.016
	60 SR	0.886 ± 0.033	0.999 ± 0.001 ^c^	0.984 ± 0.007 ^c^	0.976 ± 0.052 ^c^	-
ssGBLUP	Bw	-	0.347 ± 0.050	0.359 ± 0.017	0.792 ± 0.055	0.789 ± 0.017
	ST	0.744 ± 0.091	-	0.784 ± 0.009	0.983 ± 0.002 ^c^	0.930 ± 0.007 ^c^
	36 SR	0.427 ± 0.032	0.997 ± 0.003 ^c^	-	0.995 ± 0.009 ^c^	0.935 ± 0.016 ^c^
	SS_50_	0.906 ± 0.032 ^c^	0.999 ± 0.001 ^c^	0.998 ± 0.002 ^c^	-	0.979 ± 0.001 ^c^
	60 SR	0.891 ± 0.023	0.999 ± 0.002 ^c^	0.962 ± 0.014 ^c^	0.979 ± 0.001 ^c^	-

Notes: Bw: body weight; ST: the time from the initiation of toxic pellet feeding at 0 h to the death of the shrimp; 36 SR: the survival rate of each family at 36 h; SS_50_: the survival rate of each family when the mortality within the strain reached 50%; and 60 SR: the survival rate of each family at 60 h. Phenotypic correlation (above the diagonal) and genetic correlation (under the diagonal). ^c^: estimates are not significantly different from one (*p* > 0.05).

**Table 6:** Predictive accuracy and predictive bias of different traits under various methods.

Method	Strain	MK					GK				
	Trait	Bw	ST	36 SR	SS_50_	60 SR	Bw	ST	36 SR	SS_50_	60 SR
pBLUP	Acc	0.420	0.328	0.319	0.314	0.302	0.491	0.314	0.272	0.261	0.308
	Bias	1.012	1.015	1.026	1.016	1.006	1.004	1.006	1.027	1.021	1.024
ssGBLUP	Acc	0.420	0.328	0.319	0.314	0.302	0.492	0.315	0.272	0.262	0.308
	Bias	1.012	1.015	1.026	1.017	1.006	1.004	1.011	1.027	1.021	1.024

Notes: Bw: body weight; ST: the time from the initiation of toxic pellet feeding at 0 h to the death of the shrimp; 36 SR: the survival rate of each family at 36 h; SS_50_: the survival rate of each family when the mortality within the strain reached 50%; and 60 SR: the survival rate of each family at 60 h. Acc: predictive accuracy; Bias: predictive bias.

## Data Availability

The original contributions presented in this study are included in the article; further inquiries can be directed to the corresponding author.

## References

[1] (2024). FAO FishStat Database. https://www.fao.org/fishery/statistics-query/en/global_production/global_production_quantity.

[2] Zhang W.Q. (1990). Biological introduction of the world’s important aquaculture species—White leg Shrimp in South America. Mar. Sci..

[3] MOA (Ministry of Agriculture and Rural of the People’s Republic of China) (2024). China Fishery Statistical Yearbook.

[4] Kumar V., Roy S., Behera B.K. (2021). Acute Hepatopancreatic Necrosis Disease (AHPND): Virulence, Pathogenesis and Mitigation Strategies in Shrimp Aquaculture. Toxins.

[5] Lee C.T., Chen I.T., Yang Y.T. (2015). The opportunistic marine pathogen *Vibrio parahaemolyticus* becomes virulent by acquiring a plasmid that expresses a deadly toxin. Proc. Natl. Acad. Sci. USA.

[6] Tran L., Nunan L., Redman R.M. (2013). Determination of the infectious nature of the agent of acute hepatopancreatic necrosis syndrome affecting penaeid shrimp. Dis. Aquat. Org..

[7] Kongrueng J., Yingkajorn M., Bunpa S. (2015). Characterization of Vibrio parahaemolyticus causing acute hepatopancreatic necrosis disease in southern Thailand. J. Fish Dis..

[8] Navaneeth K.A., Bhuvaneswari T., Rajan J.J.S. (2020). Characterization of *Vibrio parahaemolyticus* isolates from shrimp farms of Southeast coast of India with special reference to acute hepatopancreatic necrosis disease (AHPND) status. Aquaculture.

[9] Tang K.F., Bondad Reantaso M.G., Arthur J.R. (2020). Shrimp Acute Hepatopancreatic Necrosis Disease Strategy Manual.

[10] Cock J., Gitterle T., Salazar M. (2009). Breeding for disease resistance of Penaeid shrimps. Aquaculture.

[11] Moss S.M., Moss D.R., Arce S.M. (2012). The role of selective breeding and biosecurity in the prevention of disease in penaeid shrimp aquaculture. J. Invertebr. Pathol..

[12] Nielsen H.M., Sonesson A.K., Yazdi H. (2009). Comparison of accuracy of genome-wide and BLUP breeding value estimates in sib based aquaculture breeding schemes. Aquaculture.

[13] Nirea K.G., Sonesson A.K., Woolliams J.A. (2012). Strategies for implementing genomic selection in family-based aquaculture breeding schemes: Double haploid sib test populations. Genet. Sel. Evol..

[14] Hayes B.J., Bowman P.J., Chamberlain A.J., Goddard M.E. (2009). Invited review: Genomic selection in dairy cattle: Progress and challenges. J. Dairy Sci..

[15] Legarra A., Christensen O.F., Aguilar I. (2014). Single step, a general approach for genomic selection. Livest. Sci..

[16] Aguilar I., Misztal I., Johnson D.L. (2010). Hot topic: A unified approach to utilize phenotypic, full pedigree, and genomic information for genetic evaluation of Holstein final score. J. Dairy Sci..

[17] Misztal I., Legarra A., Aguilar I. (2009). Computing procedures for genetic evaluation including phenotypic, full pedigree, and genomic information. J. Dairy Sci..

[18] Yoshida G.M., Carvalheiro R., Rodríguez F.H. (2019). Single-step genomic evaluation improves accuracy of breeding value predictions for resistance to infectious pancreatic necrosis virus in rainbow trout. Genomics.

[19] Liu J., Yang G., Kong J. (2020). Using single-step genomic best linear unbiased prediction to improve the efficiency of genetic evaluation on body weight in *Macrobrachium rosenbergii*. Aquaculture.

[20] Sae-Lim P., Kause A., Lillehammer M. (2017). Estimation of breeding values for uniformity of growth in Atlantic salmon (*Salmo salar*) using pedigree relationships or single-step genomic evaluation. Genet. Sel. Evol..

[21] Fu Q., Sun K., Sui J. (2023). Comparisons and genetic assessments of WSSV resistance and growth in strain cross of *Litopenaeus vannamei*. Aquac. Rep..

[22] Tan J., Luan S., Cao B.X. (2019). Evaluation of genetic parameters for reproductive traits and growth rate in the Pacific white shrimp *Litopenaeus vannamei* reared in brackish water. Aquaculture.

[23] https://kns.cnki.net/kcms2/article/abstract?v=EKYfHJ8l29gheTwCWoNdLMhOnnqHilPRrNk-uV_jy0YAXNizeoV2hIz09_OsN2-aQkAQKYi3UOyK6MvsDV6JITxfOy50R8oTT9BvLLhCJzp7YVMquoKGQ5dQkJjXeOYCIp9he5mdWHPf0cvzyeyieSyuVnvxAjjESjvG5kb_mY8TJw4KGoZ6UADzqYKcaPeK&uniplatform=NZKPT.

[24] (2024). FAO FishStat Database. https://kns.cnki.net/kcms2/article/abstract?v=EKYfHJ8l29j3OUMvsXJGNExUIvi9J5XXywor_quRN1KuSVJWl4RSeWNW-jGLkb6iXlo7tyYa5fHYVKe8YRI9ddFPwwT8R7TETQpB_zHCar64lEQ7ZXBVSwqklfRC1w42Uy9s4ARlIVW-ponQslHPgSQ9rumR8QSym81R0JiygN6HlHwIQYL7mg==&uniplatform=NZKPT&language=CHS.

[25] Butler D., Cullis B., Gilmour A., Gogel B., Thompson R. (2020). ASReml-R Reference Manual Version 4.1. 0.130. VSN International Ltd..

[26] Misztal I.S., Tsuruta D.A.L. (2014). Lourenco Manual for BLUPF90 Family of Programs.

[27] Lu X., Luan S., Cao B.X. (2017). Estimation of genetic parameters and genotype-by-environment interactions related to acute ammonia stress in Pacific white shrimp (*Litopenaeus vannamei*) juveniles at two different salinity levels. PLoS ONE.

[28] Liu M.Y., Li X.P., Kong J. (2023). Application of the liquid chip “Yellow Sea Chip No. 1” in genetic evaluation of the base population with resistance to acute hepatopancreatic necrosis disease in *Litopenaeus vannamei*. J. Fish. China.

[29] Hernández-Ruíz H., Montaldo H.H., Bustos-Martínez J. (2020). Heritability and genetic correlations for infectious hypodermal and hematopoietic necrosis virus load, body weight at harvest, and survival rate in Pacific white shrimp (*Litopenaeus vannamei*). J. World Aquac. Soc..

[30] Sun K., Li X.P., Sui J. (2022). Evaluation of genetic parameters of body length, body weight and WSSV resistance of *Litopenaeus vannamei* based on microsatellite markers. J. Fish. China.

[31] Chen M.J., Kong J., Tan J. (2021). Unknown parental group effects on harvest body weight in the base population of *Litopenaeus vannamei*. J. Fish. Sci. China.

[32] Luan S., Luo K., Chai Z. (2015). An analysis of indirect genetic effects on adult body weight of the Pacific white shrimp *Litopenaeus vannamei* at low rearing density. Genet. Sel. Evol..

[33] Trang T.T., Hung N.H., Ninh N.H. (2019). Selection for improved white spot syndrome virus resistance increased larval survival and growth rate of Pacific Whiteleg shrimp, *Liptopenaeus vannamei*. J. Invertebr. Pathol..

[34] Sui J., Sun K., Kong J. (2024). Estimation of genetic parameters for growth and wssv resistance traits in *Litopenaeus vannamei*. Animals.

[35] Benzie J., Kenway M., Trott L. (1997). Estimates for the heritability of size in juvenile *Penaeus monodon* prawns from half-sib matings. Aquaculture.

[36] Dai P., Zheng J., Luan S. (2022). Estimates of heritability and genetic correlation for growth traits at harvest in redclaw crayfish, *Cherax quadricarinatus*. Aquaculture.

[37] Nguyen N.H., Khaw H.L., Ponzoni R.W. (2007). Can sexual dimorphism and body shape be altered in Nile tilapia (*Oreochromis niloticus*) by genetic means. Aquaculture.

[38] Rye M.-A., Mao I. (1998). Nonadditive genetic effects and inbreeding depression for body weight in Atlantic salmon (*Salmo salar* L.). Livest. Prod. Sci..

[39] Pante M.J.R., Gjerde B., Mcmillan I. (2002). Estimation of additive and dominance genetic variances for body weight at harvest in rainbow trout, *Oncorhynchus mykiss*. Aquaculture.

[40] Thodesen J., Rye M., Wang Y.X. (2011). Genetic improvement of tilapias in China: Genetic parameters and selection responses in growth of Nile tilapia (*Oreochromis niloticus*) after six generations of multi-trait selection for growth and fillet yield. Aquaculture.

[41] Rutten M.J., Komen H., Bovenhuis H. (2005). Longitudinal genetic analysis of Nile tilapia (*Oreochromis niloticus* L.) body weight using a random regression model. Aquaculture.

[42] Wang J., Bai Y., Zou X., Li C., Yang J., Ke Q. (2023). First Genomic Prediction of Single-Step Models in Large Yellow Croaker. Mar. Biotechnol..

[43] Dai P., Luan S., Lu X., Luo K., Cao B., Meng X. (2017). Genetic evaluation of feed efficiency in the breeding population of *Fenneropenaeus chinensis* “Huanghai No. 2” using phenotypic, pedigree and genomic information. Aquac. Int..

[44] Liu M., Li X., Kong J., Meng X., Luo K., Sui J. (2025). Genomic selection of resistance to acute hepatopancreatic necrosis disease in *Litopenaeus vannamei*. Aquaculture.

[45] Garcia A.L., Bosworth B., Waldbieser G., Misztal I., Tsuruta S., Lourenco D.-A. (2018). Development of genomic predictions for harvest and carcass weight in channel catfish. Genet. Sel. Evol..

[46] Van Grevenhof E.M., Van Arendonk J.A., Bijma P. (2012). Response to genomic selection: The Bulmer effect and the potential of genomic selection when the number of phenotypic records is limiting. Genet. Sel. Evol..

[47] Boligon A.A., Long N., Albuquerque L.G.D., Weigel K.A., Gianola D., Rosa G.J.M. (2012). Comparison of selective genotyping strategies for prediction of breeding values in a population undergoing selection. J. Anim. Sci..

[48] Dai P., Kong J., Liu J. (2020). Evaluation of the utility of genomic information to improve genetic evaluation of feed efficiency traits of the Pacific white shrimp *Litopenaeus vannamei*. Aquaculture.

[49] Vallejo R.-L., Leeds T.D., Gao G. (2017). Genomic selection models double the accuracy of predicted breeding values for bacterial cold water disease resistance compared to a traditional pedigree-based model in rainbow trout aquaculture. Genet. Sel. Evol..

[50] Yang J., Benyamin B., Mcevoy B.P. (2010). Common SNPs explain a large proportion of the heritability for human height. Nat. Genet..

[51] Muir W.M. (2007). Comparison of genomic and traditional BLUP-estimated breeding value accuracy and selection response under alternative trait and genomic parameters. J. Anim. Breed. Genet..

[52] Kudinov A.A., Nousiainen A., Koskinen H. (2024). Single-step genomic prediction for body weight and maturity age in Finnish rainbow trout (*Oncorhynchus mykiss*). Aquaculture.

[53] Legarra A., Reverter A. (2018). Semi-parametric estimates of population accuracy and bias of predictions of breeding values and future phenotypes using the LR method. Genet. Sel. Evol..

[54] Vallejo R.L., Pietrak M.R., Milligan M.M. (2024). Genetic architecture and accuracy of predicted genomic breeding values for sea lice resistance in the St John River aquaculture strain of North American Atlantic salmon. Aquaculture.

[55] Onogi A., Ogino A., Komatsu T., Simizu K., Kurogi K., Yasumori T., Togashi K., Iwata H. (2014). Genomic prediction in Japanese Black cattle: Application of a single-step approach to beef cattle. J. Anim. Sci..

[56] Wang Q., Yu Y., Zhang Q. (2019). Evaluation on the genomic selection in *Litopenaeus vannamei* for the resistance against *Vibrio parahaemolyticus*. Aquaculture.

[57] Lyu D., Yu Y., Zhang Q. (2020). Estimating genetic parameters for resistance to *Vibrio parahaemolyticus* with molecular markers in Pacific white shrimp. Aquaculture.

[58] Liu Y., Luan S., Liu M.Y. (2023). Genomic prediction accuracy analysis of AHPND resistance genome prediction in *Litopenaeus vannamei* using SNP panels with different densities. J. Fish. China.

[59] Huang G.X., Li X.P., Tian J.T. (2024). Estimation of genetic parameters for growth and acute hepatopancreatic necrosis resistance in different strains of *Litopenaeus vannamei*. Prog. Fish. Sci..

[60] Campos-Montes G.R., Caballero-Zamora A., Montaldo H.H. (2020). Genetic (co)variation in resistance of Pacific white shrimp *Litopenaeus vannamei* to acute hepatopancreatic necrosis disease (AHPND) and white spot syndrome virus (WSSV) in challenge tests. Aquaculture.

[61] Coman G.J., Arnold S.J., Wood A.T. (2010). Age: Age genetic correlations for weight of Penaeus monodon reared in broodstock tank systems. Aquaculture.

[62] Luan S., Luo K., Ruan X.H. (2013). Genetic parameters and genotype by environment interaction for body weight and survival of pacific white shrimp *Litopenaeus vannamei*. Oceanol. Et Limnol. Sin..

[63] Argue B.J., Arce S.M., Lotz J.M. (2002). Selective breeding of Pacific white shrimp (*Litopenaeus vannamei*) for growth and resistance to Taura Syndrome Virus. Aquaculture.

[64] Feng Y.P. (2017). Estimation of Genetic Parameters for White Spot Syndrome Virus (WSSV) Resistance Traits in Litopenaeus vannmei and Comparison of Resistance to WSSV between Litopenaeus vannamei and Fenneropenaeus chinensis.

[65] Li Z.X., Wang W.J., Hu Y.L. (2018). Estimation of Genetic Parameters for Four-Month Old Turbot (*Scophthalmus maximus*) Resistance to Ammonium. Periodical Ocean. Univ. China.

